# The ongoing impact of COVID-19 on adult cardiac surgery and suggestions for safe continuation throughout the pandemic: a review of expert opinions

**DOI:** 10.1177/02676591211013730

**Published:** 2021-05-13

**Authors:** Kirstie Kirkley, Umberto Benedetto, Massimo Caputo, Gianni D Angelini, Hunaid A Vohra

**Affiliations:** Department of Cardiac Surgery/Cardiovascular Sciences, Bristol Heart Institute, University of Bristol, Bristol, UK

**Keywords:** COVID-19, adult cardiac surgery, pandemic, elective procedures

## Abstract

**Objectives:**

To establish the impact of the COVID-19 pandemic on adult cardiac surgery by reviewing current data and use this to establish methods for safely continuing to carry out surgery.

**Methods:**

Conduction of a literature search via PubMed using the search terms: ‘(adult cardiac OR cardiothoracic OR surgery OR minimally invasive OR sternotomy OR hemi-sternotomy OR aortic valve OR mitral valve OR elective OR emergency) AND (COVID-19 or coronavirus OR SARS-CoV-2 OR 2019-nCoV OR 2019 novel coronavirus OR pandemic)’. Thirty-two articles were selected.

**Results:**

Cardiac surgery patients have an increased risk of complications from COVID-19 and require vital finite resources such as intensive care beds, also required by COVID-19 patients. Thus reducing their admission and potential hospital-acquired infection with COVID-19 is paramount. During the peak, only emergencies such as acute aortic dissections were treated, triaging patients according to surgical priority and cancelling all elective procedures. Screening and 2-week quarantine prior to admission were essential changes, alongside additional levels of PPE. Focus was on reducing length of stay and switching to day-cases to reduce post-operative transmission risk, whilst several hospitals adopted ‘hot’ and ‘cold’ operating theatres for covid-confirmed and covid-negative patients.

**Conclusions:**

This paper suggests a ‘CARDIO’ approach for reintroducing elective procedures: ‘Care, Assess, Re-Evaluate, Develop, Implement, Overcome’; prioritising the mental and physical health of the workforce, learning from and sharing experiences and objectively prioritising patients to improve case load.

## Introduction

Coronavirus Disease 2019 (COVID-19), the infectious disease and resulting pneumonia attributed to the 2019 novel coronavirus (2019-nCoV), (also, SARS-CoV-2) ‘has presented the most significant global health emergency to date’.^1^ Following a cluster of ‘pneumonia of unknown aetiology’ reported in Wuhan, China, in December 2019, the virus has spread globally, being declared a pandemic by the World Health Organisation (WHO) on 11th March 2020.^2^ Now, over a year on, with a catastrophic second wave, there have been over 120 million cases and almost 3 million deaths globally, 125 thousand of which were in the UK, the 5th highest total in the world.^3^ Far more contagious than its SARS and MERS counterparts, with an estimated transmission rate of around 2–2.5, global objectives have been to reduce the spread and avoid overwhelming healthcare systems whilst a vaccination schedule can be established and rolled out.^3^ Early evidence suggests a mortality rate of ~4%, with 5% of cases requiring admission to an intensive care unit (ICU).^1^ As such, the need to identify particular risk groups and minimise hospital admission is essential for efficiently prioritising limited resources.^4^

Cardiac surgical patients not only require vital ICU resources but are also potentially in the highest risk category of complications of COVID-19.^5–8^ Considering the cancellation of elective surgeries, limited scientific evidence exists that can provide structured guidance on how to prioritise patients and resources during this time. Instead, surgeons are relying on clinical experience and collated expert opinion to shape practice.^5^ This review aims to summarise the existing literature on the impact of COVID-19 on both cardiac patients and the surgical specialty and suggest methods for safely carrying out surgical interventions despite the persistence of COVID-19 cases.

## Methods

A thorough literature search was conducted using the PubMed database with the following search thread: ‘(adult cardiac OR cardiothoracic OR surgery OR minimally invasive OR sternotomy OR hemi-sternotomy OR aortic valve OR mitral valve OR elective OR emergency) AND (COVID-19 or coronavirus OR SARS-CoV-2 OR 2019-nCoV OR 2019 novel coronavirus OR pandemic)’. From this search, we included 32 articles for our review. Further articles were identified by a cross-referencing check from the identified articles.

## Cardiovascular disease and COVID-19

From the outset, it has been apparent that mortality rates from COVID-19 strongly correlate with male sex and increasing age. Early studies show shorter durations from first symptoms to death in people aged 70 and over and the greatest fatalities in those >85.^6,7^ This is particularly concerning considering the older, male, demographic accounts for a substantial proportion of those with cardiovascular disease (CVD) and associated co-morbidities, factors also predisposing risk of COVID-19.^6–8^

This interaction appears bidirectional and multifactorial, with virulence of SARS-CoV-2 reliant on binding to the angiotensin-converting-enzyme 2, expression of which is particularly high in the cardiovascular system.^1,6^ Mortality is estimated fivefold greater in those with CVD; 35% of those with severe disease have diagnosed hypertension, 17% have coronary heart disease and those with elevated troponin levels are at greater risk than controls.^6^ Comorbidities such as diabetes mellitus could impede immune function, reducing ability to clear the virus.^1^ Comparatively, circulating cytokines in COVID-19 could increase incidence of acute coronary syndromes in those with pre-existing CVD, whilst the severest pneumonia in those with such risk factors, causes ventricular strain and reduces function.^1,6^ Thus, it is apparent that COVID-19 and CVD have a bidirectional, mortality-perpetuating relationship wherein the long-term consequences of COVID-19 are worsened by and cause worsening of, CVD and other related comorbidities.

## Cardiac surgery and COVID-19

The COVID-19 pandemic has undoubtedly impacted all aspects of healthcare; diverting resources and overwhelming hospital beds with infected patients. Specialty-wide, cardiac surgery requires the most intensive care beds, a demand for which has not reduced despite the cancellation of elective surgeries.^4^ Patients over 70 years old, and those with CVD are deemed ‘moderate risk’ and, as such, must be especially vigilant about social distancing and remaining home, however, naturally, are also those likely to require definitive surgical treatment for cardiac conditions.^9,10^ Therefore, a unique ethical dilemma arises in deciding which patients to admit to hospital. Surgeons have been forced to impose constant risk: benefit stratification for patients whom delayed surgery could be fatal, but are also potentially at the highest risk of severe complications of COVID-19, contraction of which increases exponentially with leaving home.^4,5,11^

## To treat or not to treat?

**Figure 1.**
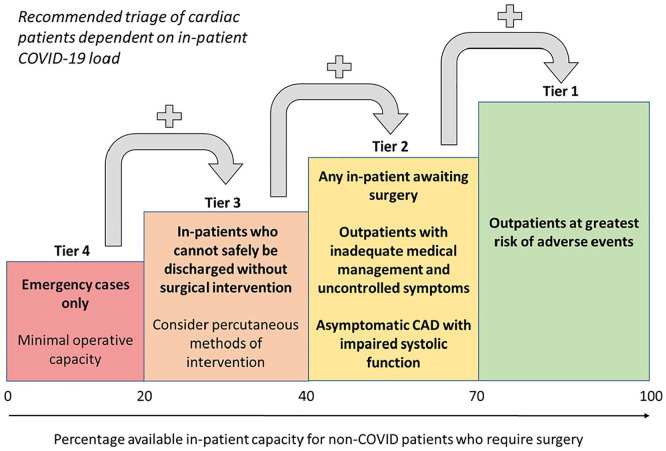
A graphic representation of the triage approach to cardiac surgery during the pandemic. The ‘traffic light’ colour scheme indicates the reduction in pressures on services, reflected in an increase in in-patient capacity and thus availability for cardiac surgery. Tier 4 indicates only 0%–20% availability for non-COVID patients; only emergency cases can be carried out. Each tier upwards offers a stepwise approach.

Currently, no concrete recommendations exist regarding the practice of cardiac surgery during the pandemic and, as such, expert opinion and multidisciplinary team (MDT) meetings have guided proceedings on a case-by-case basis.^5^ The Society of Cardiothoracic Surgery (SCTS) published advice on undertaking procedures, structured in a ‘tiered approach’ based on the inpatient COVID-19 burden, summarised in Figure 1.^4^ In the most severe circumstances, when COVID-19 inpatients account for >80% of patient load, capacity allows only for emergencies. However, as this is reduced, a wider range of essential services are considered. For example, tier 2 accounts for in-patients awaiting surgery *and* outpatients with conditions such as symptomatic very severe aortic stenosis (AS), critical coronary artery disease (CAD) and aortic aneurysms >6 cm.^4^ Across all tiers, asymptomatic outpatients are lowest in priority.^4^

Of course, in-patient COVID-19 burden is not the sole consideration for surgery. A nationwide survey of cardiothoracic surgeons suggested that, patients with confirmed or suspected COVID-19, presenting with acute type A dissection, should only be operated on in best chances of survival **and** with no active symptoms of coronavirus, such as cough or fever.^5^ Similarly, aortic and mitral valve operations should only occur in selected cases with symptomatic presentations of valve disease.^5^ Finally, emphasis is placed on the dynamic nature of the triage approach to cardiac surgery; medical professionals must be willing to constantly re-evaluate their protocols should the COVID-19 burden increase again or requirement arise for resources to be diverted elsewhere.^12^

## The impact of COVID-19 on cardiac surgery practice

### Pre-operatively

#### Screening and testing

Aside from the decision on whether to undertake surgery, potentially one of the biggest changes to pre-operative practice is the need for adequate testing. Guidance from the SCTS suggests telephone screening appointments and the nasopharyngeal nucleic acid amplification test (NAAT).^11^ Quarantine and pre-operative screening has relied on evidence of symptoms or a positive test, however, whilst false positives from NAAT are rare, false negative rates could be as high as 16%.^11,13–16^ The sensitivity of NAAT is thought to range between 57.9% and 94.6% and, considering that potentially up to 60% of patients are asymptomatic of coronavirus, this has proved particularly problematic for evaluating risk and reducing disease spread.^13–16^ Discrepancies remain regarding the use of pre-operative thoracic computerised tomography (CT) as a screening tool, wherein sensitivity is only increased in patients symptomatic of COVID-19 or those with a positive NAAT test.^10,13–18^ CT may still be used in emergency cases to maintain COVID-free hospitals and prevent the life-threatening implications of an outbreak in such areas.^18–20^ Likely, a combination of NAAT, CT and antibody detection would be optimal for reducing false negative rates, however is impractical and costly. Debate still remains as to whether all cases should be assumed positive despite a negative swab, requiring all patients to quarantine for 2 weeks prior to admission.^13–20^ Since the presence of coronavirus symptoms, or positive test, heavily influences the decision to undergo surgery, adequate screening tools are a vital pre-operative addition. They not only ensure minimum risk of transmission to other patients and crucial specialist staff; surgeons, perfusionists and critical care workers, but also a reduction in length of hospital stay and enabling beds to be vacated.

#### Consent

Another major change initiated by the pandemic is in adequately consenting patients; ensuring they are fully informed about their surgery and addressing how their experience may be different, due to the coronavirus. Understandably, patients are apprehensive about being admitted to hospital for fear of nosocomial transmission, especially since the full extent of the consequences on cardiac patients are still poorly understood.^11^ As such, patients must understand that each surgery is considered on an individual basis, to balance the risks. These are summarised, but not limited to: the risk of transmission and / or developing the COVID-19 infection; limitations within critical care and post-operative follow-up and, the risks associated with either not having surgery or delaying it further.^21^ Wherever possible, consent should be obtained by virtual means to minimise transmission, with special care taken to consider potential language barriers and disabilities, otherwise overlooked when not consenting patients in person.^21^

### Peri-operatively

**Figure 2.**
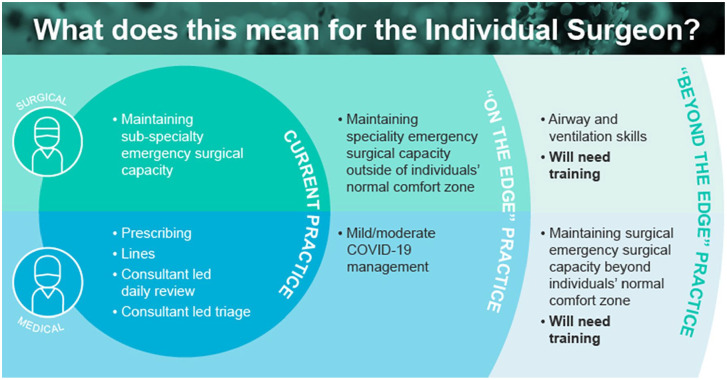
Guidance for medical and surgical teams about the expectation to meet demands outside of their normal role description. As COVID-19 pressures increase, medical and surgical practitioners may require additional training. For surgeons, this would extend to airway management and ventilation skills.^17^ Permission to reproduce from ‘Guidance for surgeons working during the COVID-19 pandemic’ by Royal College of Surgeons of England Copyright [2020] by Royal College of Surgeons.^22^ https://www.rcseng.ac.uk/coronavirus/joint-guidance-for-surgeons-v1/

Guidance from the Royal College of Surgeons (RCS) sets out two overarching themes for undertaking surgery during the COVID-19 pandemic. In short, providing the best possible outcomes for patients, triaged as in a mass casualty situation, and preservation of surgical teams.^21^ Whilst the minimum expectation is in sustaining emergency services within each specialty, surgeons may be expected to work outside of their normal jurisdiction, with potential escalation for essential additional training in ventilation and basic airway management, as summarised in Figure 2.^22^ The requirement is particularly applicable to cardiac surgeons and perfusionists who are more likely than other specialties to possess generic, transferable skills, useful for an intensive care environment. That is, experience caring for patients in the ICU; the use of mechanical and circulatory support such as extra corporeal membrane oxygenation (ECMO) and managing the potential acute and chronic cardiac complications associated with COVID-19 infection.^10,12,23^ Consequently, the disruption and modifications, purely based on workforce restructuring, have been multifold.

#### Personal protective equipment and infection control

**Figure 3.**
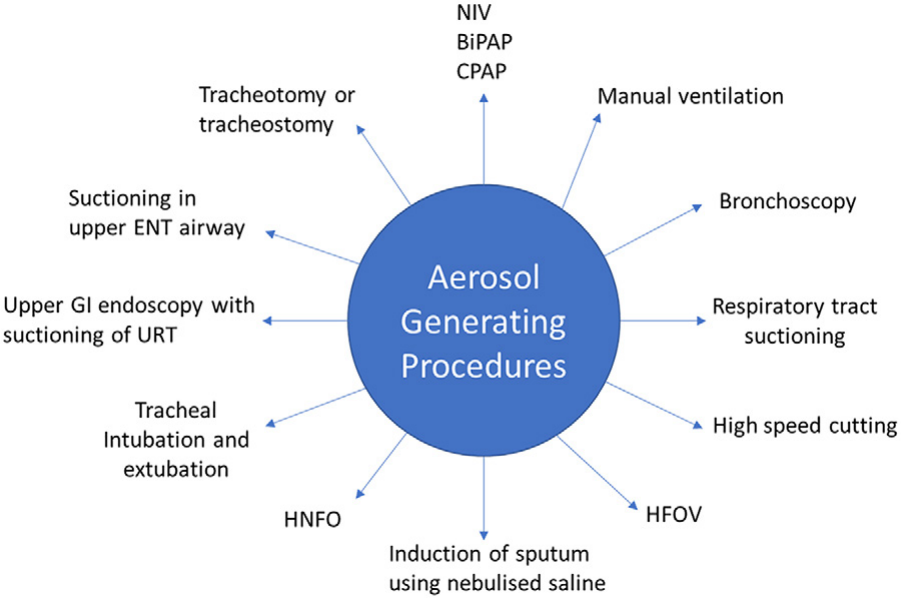
A summary of AGPs according to Public Health England. Before commencing surgery, any potential AGPs must be considered and a list conveyed to the theatre team. NIV: non invasive ventilation; BiPAP: Bi-level positive airway pressure; CPAP: continuous positive airway pressure; HFOV: high frequency oscillatory ventilation; HNFO: high flow nasal oxygen; GI: gastro-intestinal; URT: upper respiratory tract; ENT: ear, nose and throat.

**Figure 4.**
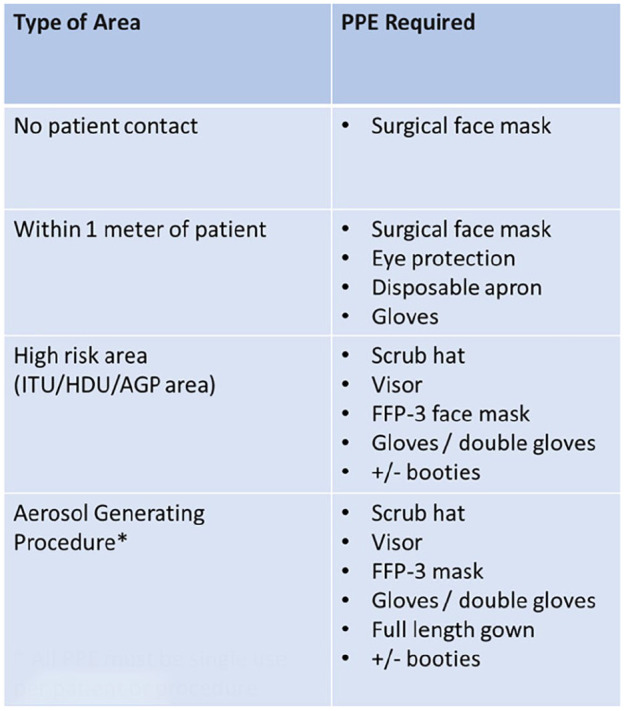
All staff must keep up to date with the PPE requirements as per each risk area; even in the absence of patient contact, a surgical face mask must be worn. In high risk areas, such as the ITU or HDU, the FFP-3 face mask and a visor is donned. In the case of any AGPs, PPE is single use per patient or procedure and a full-length gown may be added. *All PPE must be single use per patient or procedure.

The first step in both ensuring positive patient outcomes *and* preserving the surgical workforce is enforcing stringent infection control measures and use of adequate personal protective equipment (PPE). Expert opinion advocated the use of full PPE for all members of the theatre team for every procedure, irrespective of COVID-19 diagnosis.^5^ COVID-19 means every surgery poses a greater risk to both patients and staff, and as such, theatre staffing levels are minimised, however, the nature of cardiac surgery puts these teams in the highest risk category for exposure.^24^ Conduction of aerosol generating procedures (AGPs), such as intubation, extubation and potential resuscitation, alongside the need to be within 1 m of a possible or confirmed COVID-19 patient, mean the maximum levels of PPE should be donned at all times.^25^ AGPs and PPE requirements are summarised in Figures 3 and 4 respectively.^25,26^

The recommended FFP-3 mask, correctly fitted, can block an estimated 99.7% of particles, proving 100–10,000 times more effective than no mask (compared to 6× in regular masks).^18^ However, during routine but lengthy cardiac procedures, the comfort of those, such as surgeons, perfusion specialists, scrub nurses and the rest of the theatre personnel wearing full PPE, must also be considered.^23^ Ongoing analysis of expert opinion questions whether masks of such high specification are necessary. One survey suggests that 58.3% of cardiac surgeons wore the FFP-3 masks during surgery, despite only 45.8% agreeing with this practice and a notable 37.5% deeming a surgical mask sufficient.^20^ Minimal staffing adds further tensions to an already high pressure environment and full PPE is uncomfortable and cumbersome. Questions must be asked as to the impact of such extensive PPE on surgeons’ ability to communicate effectively with the rest of the team, performance fatigue and double-gloving on dexterity, the latter of which recommendations, 79% of surgeons deemed unnecessary.^15^ Again, risk: benefit stratification is implemented; the limitations and detriment caused to surgeons, and thus patients, by potentially unnecessary PPE, versus the extent of protection provided to an already limited, invaluable workforce.^18,20^

Additional measures for infection control have particularly implicated the process of anaesthestics, wherein extubation and its associated coughing creates necessity for additional precautionary measures in reducing staff exposure to COVID-19. As a minimum, disposable equipment is strongly encouraged and extubation and recovery should take place inside the theatre, a designated ‘dirty zone’, ensuring the scrub room and anaesthetic room remain uncontaminated.^27^ Additionally, switching from positive to negative pressure operating rooms (ORs) aims to extract the virus from the theatre environment and reduce dissemination.^24^ However, where this is not possible, the use of positive pressure operating rooms is discouraged: this should be turned off for at least 20 minutes, the theatre cleaned and then restarted to refresh the air before commencing another surgery.^27^

#### Organisation and hospital structure

Whilst different regions, hospitals and specialties have adopted varying approaches to tackling the logistics of undertaking surgery during the pandemic, the underlying principles are largely consistent. In short, isolation of patients who are confirmed or suspected COVID-19 positive, from those who are not, to reduce transmission and categorise risk.

In Italy, a ‘hub and spoke’ hospital system has been adopted, 16 hospitals closed to remain infection-free, leaving four ‘hub’ hospitals to perform cardiac procedures.^10^ The ‘hubs’ will dedicate one operating room for COVID-positive patients and ‘spokes’ will help triage patients to be referred, with cases shared amongst ‘hub’ surgeons.^10^ Similarly, the ‘Pan London Emergency Cardiac Surgery’ (PLECS) system triages patients according to risk, referring accordingly to the Bart’s Heart Centre or Harefield Hospital, which have allocated beds specifically for cardiac surgery patients.^19,28^ This allows those with emergencies in the London area, such as acute aortic dissections, to be prioritised for urgent intervention and creates an efficient system for deciding when and where patients require treatment. In other areas however, such as cities with only one specialised heart unit, ‘hot’ and ‘cold’ ORs and theatre teams have been designated. ‘Hot’ ORs, for those with suspected or confirmed COVID-19 patients, should: have negative pressure systems where possible; minimise team size and entrance/exit of the OR during surgery. Theoretically, this reduces disruption of laminar flow which could spread particles outside of the OR.^24^ Emergency cases should be treated in hot ORs due to insufficient time to determine COVID status.^24^ At the absolute least, hospitals have dedicated ‘clean’ and ‘dirty’ areas of the operating room, with a specified list of staff allowed in each area.^27^

#### Management and staffing

Staff restructuring in the pandemic has also been enforced in a ‘top-down’ approach, enabling the co-ordination of vital surgeries, despite the given circumstances. With cardiac surgeons and perfusionists being diverted to work in critical care, other portions of the workforce shielding after experiencing symptoms and a need to have contingency plans in place to account for resource availability and supply chain issues, doctors are expected to be managers *and* clinicians. NHS Guidance advises the designation of a ‘lead consultant’, excused from clinical duties to oversee crisis management, co-ordinate patient flow and manage stock of PPE.^25^ Inadequate staff, beds or stock can quickly hinder efficiency and as such, leadership frameworks and clear team structuring are crucial for high-pressure situations such as these and ensuring both the best outcomes for patients and the protection of vital, limited specialist staff such as perfusionists, cardiac surgeons, anaesthetists and other team members.^10,19,28,29^

#### Shift in surgical strategy

Emphasising the importance of reducing the length of stay (LOS) in hospital, guidance has promoted the shift towards day cases, managing only those who are safer in hospital than at home due to their risk of adverse cardiac events.^29^ This includes, but is not exclusive to, presentation of acute aortic dissection, patients with symptomatic AS or those requiring coronary artery bypass grafting which cannot be treated by medical means.^23^ A reduction in LOS has also been largely dependent on cross-specialty communication, with cases being transferred to cardiology to avoid surgical intervention.^23^ The general consensus has been to adopt non-surgical or conservative approaches such as transcatheter aortic valve implantation (TAVI) or percutaneous coronary intervention (PCI) wherever possible, due to the highest mortality rates associated with the peri and post-operative timeframe, keeping patients safe but also at home.^19,24^ Mortality rates for COVID-19 patients undergoing general surgery have shown to be as high as 20%, however, the rate of unnecessary deaths caused by the delay of time-critical cardiac operations and morbidity associated with worsening heart failure are still unquantified. Thus, the long-term effects of these short-term compromises are still largely unknown.^18,30^ Despite a positive association with reduced post-operative infection, faster recovery and thus reduced LOS, due to its relatively novel nature, there has been a decrease in minimally invasive cardiac surgery (MICS), such as minimally invasive mitral valve repair and minimally invasive mitral valve replacement during the pandemic.^18,20^ Experts agree that emergency procedures should only take place via conventional methods of full sternotomy.^18^ However, overwhelming expert consensus suggests there is no greater risk of adverse outcomes when compared to conventional methods in the peri-pandemic period and regardless of COVID-19 status.^20^ In addition, the avoidance of conventional sternotomy by using a minimally invasive approach reduces the exposure of surgeons, perfusionists, scrub nurses and other theatre staff to unnecessary additional AGPs.^13^ As such, the restriction of MICS is unnecessary.

### Post-operatively

#### Complications and consequences

The main issue with the novel 2019 coronavirus arises precisely from its nomenclature, that is, it is novel. Whilst cardiac surgeons and the affiliated teams have been forced to adapt intervention, despite knowing so little about the disease, the full extent of both the short and long term impacts is still largely uncertain. For example, surgeons are seeing post-operative complications in COVID-19 patients which are usually rare, such as acute biological leaflet thrombosis resulting in moderate haemorrhagic pericardial effusion, due to post-operative COVID-19 infection.^30^ This, following treatment for critical aortic valve stenosis with a biological aortic valve replacement.^30^ Consequently, there is currently a need to constantly evolve and use clinical judgement to guide decision-making outside of the usual highly-regulated practice. COVID-19 has proven extremely pro-coagulable, this creates an additional challenge for perfusionists operating heart-lung machines and also a need to reconsider the use of anti-coagulants in patients for whom these would not have been usual practice.^30^ The risk of acute respiratory distress syndrome (ARDS) is also potentially greater in COVID-19 patients undergoing surgery which requires cardiopulmonary bypass (CPB), due to the associated pro-inflammatory response with CPB and increase of circulating cytokines from COVID-19.^14^ As such, off-pump alternatives may need to be considered.^14^ Concern also arises, that, in the reducing the number of cardiac surgeries, and the need to work within the ICU, that cardiac surgeons may deskill, illustrating that the long term effects for clinicians could be as uncertain as those for patients.^18,20,23^

#### Length of hospital stay and follow-up

Appropriate and efficient planning is required to ensure that LOS is minimised, in all aspects, but especially in critical care.^29^ Guidance states that only patients who are more at risk by going home should remain in hospital, with other urgent, but stable patients, remaining at home until their surgery is due.^29^ The result, a reduction in pre-operative waiting times, allowing efficient pre-planning of discharge for priority patients, crucial in maintaining patient flow and reducing LOS. The switch to day cases, percutaneous interventions and referrals to cardiology, wherever possible, has also been paramount in instigating this change, however, where this is not possible, clinicians must be prepared to adopt a dynamic approach, if prolonged hospital stays are required after surgery.^29^ As suggested in the PLECS scheme, surgeons must be willing to repatriate their patients, in order to keep them safe and reduce their potential COVID-19 exposure.^19,26^

Naturally, post-operative follow-up has also had a limited place in the hospital, with surgeons adopting new measures to create socially-distanced checkups. Follow-up clinics and MDT meetings occur by video or telephone call, avoiding unnecessary attendees in hospital.^23,29^ However, the decision should always be made by a senior team, when considering the possibility of a virtual clinic. Further suggestions encourage efforts to reduce the invasive nature of certain follow-up appointments, minimising patients’ return to hospital and exposure of staff to AGPs. For example, in heart and lung transplant patients, non-invasive strategies to determine rejection should be used; gene expression profiling and home spirometry, rather than biopsies and bronchoscopies.^21^ This could be life-saving in preventing such immunosuppressed patients from a potentially fatal COVID-19 diagnosis.^9,21^

## How can we safely perform cardiac surgery during the pandemic?

**Figure 5.**
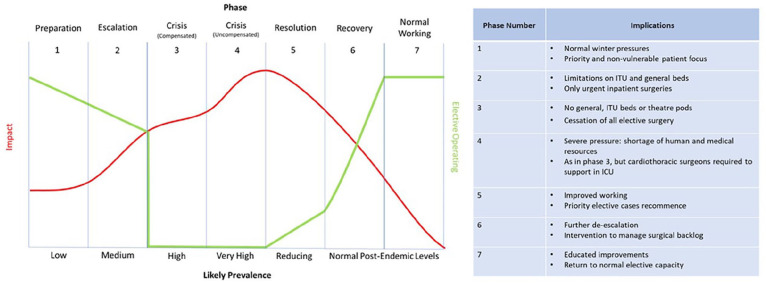
The graph demonstrates the increasing pressure of the virus as prevalence increases, corresponding with the decrease of elective procedures. The trajectory predicts that as virus prevalence decreases and resolution and recovery begin, elective procedures can begin to be re-introduced. However, it will take some time before a ‘normal service’ can be resumed.

Over the 12-week peak, at a rate of 72.3%, the pandemic has caused an estimated 28,404,603 surgeries to be cancelled worldwide, leaving costs; social, economic and environmental, in its wake.^31^ Overall, the methodology in deciding which patients to treat during the pandemic, can also be applied to prioritising the patient backlog and allowing the continuation of surgery despite ongoing coronavirus cases. The dynamic triage system and dependence on COVID-19 patient burden, allows for principles adaptable to individual areas and potential future peaks.^4^ Development of guidance by the NHS suggests management strategies according to a predicted trajectory of pandemic ‘phases’, stratifying the re-introduction of elective surgery.^29^ This is summarised by the graph in Figure 5, demonstrating the impact between phase and prevalence on hospitals and elective surgeries.^29^

In order to safely carry-out surgical procedures despite ongoing coronavirus cases, each hospital must be aware of their individual resources and the pandemic climate within their area.^32^ Cases must continue to be prioritised strategically to catch-up time that has been lost, without taking on too much, too soon.^32^ At best, if surgical workload is increased by 20%, it could take at least 45 weeks to address the backlog of cases which have built-up over the 12-week period.^31^ However, clearly, if such a ‘catch-up’ system is to be implemented, there are many considerations; not least the financial cost, at an estimated £2 billion, but physically finding the time to address the necessary percentage increase in surgical volume.^31^

To continue operating on cardiac patients and account for the backlog of cases, several things must be carefully considered: the potential delivery of a 7-day service; provisions to re-train colleagues for any loss of skill during the pandemic and methods of addressing the mental health of the surgical workforce.^23,31^ It would be negligent to assume that this pandemic has only taken a physical toll on cardiac surgery teams; the everyday stresses of complex pathology, critically unwell patients and finite resources have been amplified exponentially during this time, not accounting for the loss of patients, family members and colleagues. Thus, the ramping up of surgery must have the physical and mental health of surgical teams at the core, with tailored systems in place to help share the load. Whilst patient safety is paramount, theatre staff must continue to be stringent with use of PPE, especially during AGPs, in order to protect themselves. To assist this, the designation of a ‘lead consultant’ could be useful for ensuring sufficient stock of vital supplies. In turn, the effect of the physical protection of staff is multifold; preventing spread and thus protecting patients, but also protecting the mental health and morale of the theatre team. The need to isolate reduces already sparse staffing and so minimising this and subsequently burn-out of the remaining team is crucial for sustainability.^14,23,31^

The SCTS suggests a ‘CPR’ approach, a strategic and thus unbiased method of tackling the situation – collaborate, prioritise and re-evaluate.^11^ With this in mind, surgeons must work together to maximise efficiency against an increased case load, select the sickest patients first and be willing to monitor the situation accordingly, should change need to be implemented.^11^ Clearly this is a time for everyone to work together for the good of the system, rather than personal advancement. As such, minor consolation has been provided by the temporary cessation of surgeon-specific mortality, to avoid penalty for carrying-out usually highly regulated surgeries, with evidence-supported mortality rates, during these unpredictable circumstances, preventing the complete standstill of cardiac surgery.^5^ It is impossible to estimate the ripple effect this pandemic will have; the delay of cardiac surgery may not only worsen disease pathology, but quality of life for those lower down the priority list, with life-limiting, rather than life-critical conditions.^14,31^ It may also reduce patients’ ability to work whilst waiting for their definitive surgical treatment, times for which will only increase.^31^ More than ever, the importance of multidisciplinary teams must be at the forefront of the provision of care. The continuation of cardiac surgery cannot be a specialty-specific initiative, instead, must incorporate many aspects of healthcare systems, with strong foundations in teamwork, flexibility and selflessness.

**Figure 6.**
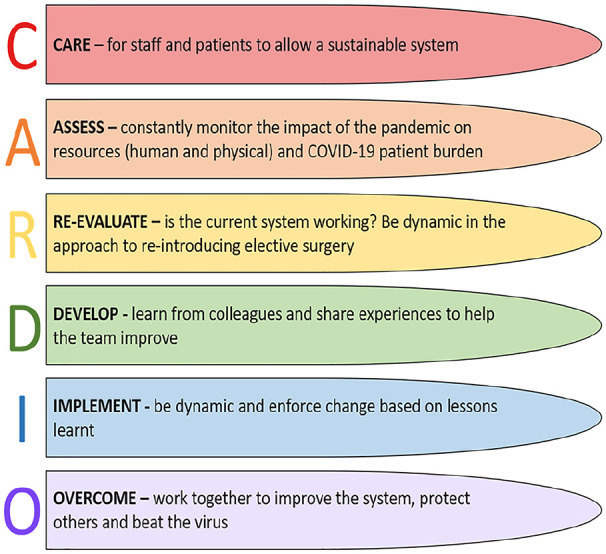
Guidance based on collated literature as to how elective surgery should be re-introduced as the pandemic burden is decreased. Focus is placed on flexibility and teamwork to provide a sustainable system which does not cause burn-out.

Collating expert opinions, any increase in workload must, first and foremost, account for the surgical workforce, if the system is to be sustainable and, not least, physically capable of caring for patients. The surgical team cannot tackle this caseload without adequate support and systems to rely on under pressure. Therefore, as COVID-19 burden falls and surgery load is allowed to increase, the following ‘CARDIO’ framework is suggested: ‘Care, Assess, Re-evaluate, Develop, Implement, Overcome’ and is laid-out in Figure 6. This is not a problem for the individual, but one solved by the improvements only seen in learning, sharing and developing from others, as an entire healthcare team.

## Conclusion

The COVID-19 pandemic has posed challenges, both human and physical, over the previous months, which no-one across the world could have predicted; the full extent of which is still waiting to unravel. Once, the epitome of medical practice, clinicians are now being forced to find ways to *avoid* human contact and postpone procedures they would be otherwise compelled to carry-out. Cardiac surgeons have been pioneers on the frontline, valiantly adapting and offering their skillset to help their colleagues in ITU, abandoning their usual practice to prioritise those most in need. This has undoubtedly had a profound negative influence on practice, however there is evidently hope that this shall return to normal, in the future. Important lessons can be learned from the ways in which different specialties have had to adapt, and how they will continue to adapt, as measures are taken to catch-up lost time. We must continue to reflect on how far we have come and maintain hope that, even though there’s still a long way to go, vast progress has been made and adaptations and successes must be recognised and commended.
